# Patterns of Risk of Cancer in Patients with Metal-on-Metal Hip Replacements versus Other Bearing Surface Types: A Record Linkage Study between a Prospective Joint Registry and General Practice Electronic Health Records in England

**DOI:** 10.1371/journal.pone.0065891

**Published:** 2013-07-04

**Authors:** Arief Lalmohamed, Alexander J. MacGregor, Frank de Vries, Hubertus G. M. Leufkens, Tjeerd P. van Staa

**Affiliations:** 1 Division of Pharmacoepidemiology and Clinical Pharmacology, Utrecht Institute of Pharmaceutical Sciences, Utrecht University, Utrecht, The Netherlands; 2 Department of Clinical Pharmacy, University Medical Center Utrecht, Utrecht, The Netherlands; 3 Norwich Medical School, University of East Anglia, Norwich United Kingdom; 4 Medical Research Council (MRC) Lifecourse Epidemiology Unit, Southampton General Hospital, Southampton, United Kingdom; 5 Department of Clinical Pharmacy and Toxicology, Maastricht University Medical Centre+, Maastricht, The Netherlands; 6 Care and Public Health Research Institute (CAPHRI), Maastricht, The Netherlands; 7 Clinical Practice Research Datalink (CPRD), Medicines and Healthcare products Regulatory Agency, London, United Kingdom; Bremen Institute of Preventive Research and Social Medicine, Germany

## Abstract

**Background:**

There are concerns that metal-on-metal hip implants may cause cancer. The objective of this study was to evaluate patterns and timing of risk of cancer in patients with metal-on-metal total hip replacements (THR).

**Methods:**

In a linkage study between the English National Joint Registry (NJR) and the Clinical Practice Research Datalink (CPRD), we selected all THR surgeries (NJR) between 2003 and 2010 (n = 11,540). THR patients were stratified by type of bearing surface. Patients were followed up for cancer and Poisson regression was used to derive adjusted relative rates (RR).

**Results:**

The risk of cancer was similar in patients with hip resurfacing (RR 0.69; 95% Confidence Interval [CI] 0.39–1.22) or other types of bearing surfaces (RR 0.96; 95% CI 0.64–1.43) compared to individuals with stemmed metal-on-metal THR. The pattern of cancer risk over time did not support a detrimental effect of metal hip implants. There was substantial confounding: patients with metal-on-metal THRs used fewer drugs and had less comorbidity.

**Conclusions:**

Metal-on-metal THRs were not associated with an increased risk of cancer. There were substantial baseline differences between the different hip implants, indicating possibility of confounding in the comparisons between different types of THR implants.

## Introduction

Total hip replacement (THR) is a highly effective procedure performed in patients with moderate to severe osteoarthritis [1]. It is now ranked among the most common surgical operations performed worldwide, with over one million procedures estimated to be carried out annually [2], [3]. Over the past few decades, metal-on-metal hip devices gained popularity, and up to recently accounted for approximately 14% of all THRs in England and Wales [4], [5]. The use of these devices, which have been associated with the widespread dissemination of metal ions including cobalt and chromium [6], has raised a number of health concerns, including the potential risk of cancer [7], [8]. The carcinogenic properties of the materials used in these hip devices have been demonstrated previously [7], [9]. Cobalt and chromium at similar concentration levels found in post mortem specimens, induce carcinomas in animal models, and in addition may increase the chance of malignant degeneration [10].

There is limited epidemiological evidence on cancer risk following metal-on-metal THR compared with other bearing surface types. Two recent studies showed no excess risk of any cancer with metal-on-metal hip devices over other hip implants [8], [11]. The majority of the epidemiological studies could not differentiate between bearing surface types (or could not make a comparison with non metal-on-metal implants), and reported somewhat conflicting findings [12]–[20]. A meta-analysis aggregating nine of these observational studies showed no increased risk of any cancer with any THR prosthesis, although they were able to detect an elevated risk of prostate and skin cancer [21].

A comparison between bearing surface types has many limitations, as there is a high probability of confounding by indication. Metal-on-metal hip replacements (in particular hip resurfacing) are generally considered in younger and healthier patients. It is important to study which patients are more likely to get certain bearing surface types, as these patient characteristics may also be associated with risk of cancer (and thereby introducing bias). Previous studies have not evaluated these differences in detail. To do so, this would require a linkage between high quality registries providing information on implant details (such as the UK National Joint Registry, NJR), and electronic health records with information on clinical confounders and outcomes (such as the UK Clinical Practice Research Datalink [CPRD]). Consequently, the objectives of this study were to evaluate patterns and timing of risk of cancer in patients with metal-on-metal THR and to identify predictors for bearing surface types, cancer and mortality.

## Methods

### Data Sources

Data for this study were obtained from the General Practice Research Database that is part of CPRD. CPRD collates the computerised medical records of general practitioners (GPs). GPs play a key role in the UK healthcare system, as they are responsible for primary healthcare and specialist referrals. Patients are semi-permanently affiliated with a practice that centralises the medical information from the GPs, specialist referrals, and hospitalizations. CPRD now contains computerised records for 650 GP practices, representing 8% of the British population. The data recorded in the CPRD include demographic information, prescription details, clinical events, preventive care provided, specialist referrals, hospital admissions, and major outcomes since 1987 [www.CPRD.com].

CPRD has now been linked individually and anonymously to other NHS datasets in England. This linkage is done by a trusted third party using information on the NHS number, date of birth, gender and postcode. The majority of patients were linked using their NHS number. At the time of this study, 250 GP practices in England participated in this linkage (about 40% of CPRD). The NHS datasets that were linked to CPRD included the National Joint Registry (NJR), which collects prospectively information on replacement surgeries. Information in this data source includes patient demographics (e.g. date of birth, sex, body mass index), operation details (e.g. hospital, anaesthetics, patient ASA grade), surgeon details (e.g. surgeon grade), procedure details (e.g. side of joint, indication for surgery), surgical approach (e.g. total replacement or resurfacing, cemented, materials used for the implants, minimally invasive techniques, computer guided surgery), intended thromboprophylaxis (both chemical and mechanical), bonegraft used, and intraoperative events. Since April 2003, the NJR has been collecting information on THRs performed in England and Wales. By the end of July 2005, the mean weekly submission of completed records had reached 2400 operations, with 99% of all hospitals on the joint registry database submitting data. The proportion of all relevant joint replacements performed in England and Wales which was included in NJR was approximately 60%. The NJR collects information for a large number of patient and surgical characteristics. In addition, CPRD was linked to the Hospital Episode Statistics (HES) that records details on the dates of hospital admission, major procedures and admission diagnoses and to death certificates (including primary and secondary cause of death). In the UK, death certificates are filled in upon death of a patient by a registered medical practitioner who has attended the patient during their last period. Death certificates are divided into two parts, containing the original underlying cause of death (part I) and diseases that may have contributed significantly to the death (part II). Diagnoses and causes of death in the HES database and causes of death registry are coded using the international classification of diseases, 10^th^ version (ICD-10). The linkage to CPRD was only available for England. Data were available for the following time periods: CPRD data (January 1987– December 2011), NJR data (April 2003– November 2010), HES data (April 1997– November 2010), death certificate data (December 2000–November 2011). For the linked datasets, the study period was defined as the latest data entry for any of the linked datasets until the earliest end of data collection. The study protocol was approved by the CPRD Independent Scientific Advisory Committee and by the Research Subcommittee of the NJR.

### Study Populations

#### Motivation study cohorts

A retrospective cohort study was conducted using CPRD, NJR and HES. Three study cohorts were identified based on a record of THR in CPRD, NJR or HES. Data on bearing surface type was only available in NJR (the coding in HES and CPRD was non-specific with respect to surface type and an analysis of anonymised free-text in CPRD only yielded limited information). The main analyses (i.e. those evaluating bearing surface type) were therefore conducted using the NJR cohort. Analyses not concerning bearing surface type were conducted using the CPRD cohort, as this data source had the largest sample size. For the latter cohort, we compared THR patients (regardless of bearing surface type) to matched referent subjects without THR surgery (see below). As this was our largest cohort, this was the only feasible way to evaluate cancer type specific rates among THR surgical patients versus matched controls. Any increase in cancer rate among this full THR cohort would be supportive that some hip implant devices may elevate the risk for cancer. To assess the consistency between these three databases, the overall risk of cancer following any THR was evaluated in all three databases.

#### Selection of THR patients and matched referent subjects

For each data source (i.e. CPRD, NJR, and HES), we selected all patients aged 18+ years who had a primary THR record in the corresponding data source within the study period. To each THR patient, up to six referent subjects without a history of THR were selected and they were matched to each THR patient by calendar time, age, sex, and practice. The index date for THR patients and matched referent subjects was the date of the primary THR. All patients had at least one year of valid data collection prior to the index date. We excluded patients with a recording of any cancer prior to the index date.

#### Follow-up

All patients were followed up from the index date until the end of the study period (i.e. the earliest end of data collection for any of the linked data sets), date of patient’s transfer out of the practice or death, whichever came first. In addition, THR patients in the NJR cohort with a bearing surface type other than metal-on-metal were censored if they had undergone conversion arthroplasty to a metal-on-metal hip device during follow-up. Not censoring these patients would lead to misclassification of the exposure (i.e. metal or non-metal), and may therefore dilute the association. We did not censor patients with the converse situation (i.e. non-metal to metal-on-metal). These patients were already exposed to metal hip implants and mutagenic processes may irreversibly lead to carcinomas, even after the conversion to non-metal hip implants.

### Outcomes

All patients were followed up for an incident record of cancer (excluding in situ and non-melanoma skin cancer) after the index date. We used three sources for cancer outcomes, including CPRD, HES and national death certificates. The analyses requiring HES or death certificates were restricted to practices participating in the linkage. Types of cancer were divided according to the possibility of being related to metal ions and included any cancer, haematological cancer (e.g. lymphoma, leukaemia, or myeloma), malignant melanoma, prostate cancer, renal cancer (bladder, ureter or kidney), or other types of cancer. Cancer was analysed using the three data sources separately, as none of these data sources were viewed to be ‘gold standard’ without any imperfections. However, findings that are consistent across the different sources are more likely to concern validated outcomes.

### Confounders

We reviewed the literature to identify potential confounders that were associated with cancer. These confounders were assessed at the index date and included the following: age, sex, calendar year, small-area socioeconomic status (for linked practices), smoking status, use of alcohol, body mass index, a history of hypertension, chronic obstructive pulmonary disease (COPD), coronary artery disease (CAD), and a prescribing in the 6 months before of NSAIDs or aspirin, oestrogen containing drugs, oral glucocorticoids, calcium/vitamin D supplements, glucose lowering agents, statins, immunosuppressive agents, bisphosphonates, renin-angiotensin-aldosterone-system (RAAS) inhibitors, platelet inhibitors, beta blockers, calcium channel blockers, diuretics and organic nitrates. Small-area socioeconomic status, smoking status, use of alcohol and body mass index were handled as categorical variables, with a separate category for missing data.

### Analyses

The following statistical analyses were conducted:

1. *Predictors of bearing surface type (NJR cohort), cancer (CPRD cohort) and all-cause mortality (CPRD cohort):* In order to assess confounding by indication, we identified predictors of bearing surface type (using the NJR cohort), in which we modelled all of the potential confounders in a logistic regression model. The outcome of interest was metal-on-metal bearing surface type (stratified by stemmed or resurfacing), compared with hip devices of other materials. In the second analysis, we identified predictors of cancer and all-cause mortality within control subjects, by modelling all potential confounders in a Poisson regression model.
2. *Bias-analysis (NJR cohort):* We evaluated risk of cancer within six months following THR surgery versus matched referent subjects, stratified by type of implant. Any altered cancer risk in this period is unlikely to be causally related to THR and most likely represents confounding by indication.
3. *Association between hip replacement (any type) and cancer risk (all three cohorts):* Poisson regression was used to estimate adjusted relative rates (RRs) for cancer incidence in the hip replacement cohorts to the referent cohorts. This analysis was performed for all three study cohorts and repeated for the three cancer data sources.
4. *Cumulative incidence of cancer (NJR cohort):* A competing risk model was used to estimate long-term risk of cancer, stratified by type of bearing surface, gender, and age. Death was considered the competing risk.
5. *Patterns and timing of cancer risk (CPRD cohort/NJR cohort):* For the first pattern analysis (CPRD cohort), we calculated RRs to compare cancer incidence in the 6–24, 25–60, and 60+ months after the index date with that in the first six months. This analysis was conducted within THR surgery patients, as well as in referent subjects, in order to compare timing and patterns. In the second analysis (NJR cohort), we analysed cancer risk over time in patients with metal-on-metal THR versus patients with other hip implant devices [22]–[25].

## Results

### Baseline Characteristics, and Predictors of Bearing Surface Types

Demographical information of THR patients in NJR and matched referent subjects is shown in Table 1 (11,540 THR patients). Patients with metal-on-metal THR were considerably younger (stemmed: 62.6 years, resurfacing: 54.5 years) compared to those with other hip implants (69.4 years). Similarly, the proportion of females was lower in individuals with metal-on-metal hip implants. Multivariate logistic regression demonstrated substantial differences in drug use and comorbidities between metal-on-metal hip replacements and non-metal hip devices. Predictors that were associated with both the bearing surface type and cancer (and/or all-cause mortality) included age, gender, smoking status, socioeconomic status, COPD, and use of oestrogen containing drugs, platelet inhibitors, and beta blockers. The mean age and gender distribution of THR cases were similar throughout all databases (46,425 THR patients in CPRD and 19,034 in HES).

**Table 1:** Baseline characteristics of patients with different types of hip replacements and matched controls (NJR cohort).

	Any bearing surface type				Stemmed metal-on-metal				Hip resurfacing				Other bearing surface			
	THR		Controls		THR		Controls		THR		Controls		THR		Controls	
Characteristic	n = 11,540		n = 69,218		n = 988		n = 5,926		n = 838		n = 5,028		n = 9,714		n = 58,264	
Follow-up time (mean, SD)	3.2	(2.1)	3.0	(2.1)	3.7	(1.7)	3.6	(1.7)	4.0	(2.0)	3.9	(2.0)	3.1	(2.1)	2.9	(2.1)
Females (%)	6,862	(59.5)	41,161	(59.5)	436	(44.1)	2,614	(44.1)	278	(33.2)	1,668	(33.2)	6,148	(63.3)	36,879	(63.3)
Age (mean, SD)	67.9	(11.0)	67.9	(11.0)	62.6	(10.9)	62.6	(10.9)	54.5	(8.2)	54.5	(8.2)	69.4	(10.4)	69.4	(10.4)
BMI (mean, SD)	28.3	(5.2)	27.4	(5.4)	28.5	(5.5)	27.5	(5.4)	28.5	(4.5)	27.8	(5.4)	28.3	(5.2)	27.3	(5.4)
Smoking status																
Non-smoker	5,647	(48.9)	30,805	(44.5)	455	(46.1)	2,417	(40.8)	416	(49.6)	1,887	(37.5)	4,776	(49.2)	26,501	(45.5)
Ex-smoker	3,193	(27.7)	17,347	(25.1)	281	(28.4)	1,425	(24.0)	170	(20.3)	1,008	(20.0)	2,742	(28.2)	14,914	(25.6)
Current smoker	1,431	(12.4)	9,931	(14.3)	151	(15.3)	1,008	(17.0)	106	(12.6)	872	(17.3)	1,174	(12.1)	8,051	(13.8)
Unknown	1,269	(11.0)	11,135	(16.1)	101	(10.2)	1,076	(18.2)	146	(17.4)	1,261	(25.1)	1,022	(10.5)	8,798	(15.1)
Medical history (%)																
Hypertension	4,963	(43.0)	26,805	(38.7)	356	(36.0)	1,860	(31.4)	172	(20.5)	949	(18.9)	4,435	(45.7)	23,996	(41.2)
COPD	462	(4.0)	3,620	(5.2)	20	(2.0)	263	(4.4)	9	(1.1)	84	(1.7)	433	(4.5)	3,273	(5.6)
Coronary artery disease	959	(8.3)	6,182	(8.9)	49	(5.0)	395	(6.7)	30	(3.6)	194	(3.9)	880	(9.1)	5,593	(9.6)
Recent prescriptions (%)																
NSAIDs	4,991	(43.2)	8,006	(11.6)	428	(43.3)	614	(10.4)	339	(40.5)	469	(9.3)	4,224	(43.5)	6,923	(11.9)
Oestrogen containing drugs	380	(3.3)	1,995	(2.9)	29	(2.9)	159	(2.7)	41	(4.9)	139	(2.8)	310	(3.2)	1,697	(2.9)
Corticosteroids	550	(4.8)	2,944	(4.3)	55	(5.6)	196	(3.3)	16	(1.9)	96	(1.9)	479	(4.9)	2,652	(4.6)
Calcium/vitamin D	968	(8.4)	4,443	(6.4)	68	(6.9)	263	(4.4)	15	(1.8)	73	(1.5)	885	(9.1)	4,107	(7.0)
Antidiabetics	636	(5.5)	5,105	(7.4)	43	(4.4)	395	(6.7)	14	(1.7)	198	(3.9)	579	(6.0)	4,512	(7.7)
Statins	3,143	(27.2)	19,100	(27.6)	205	(20.7)	1,437	(24.2)	105	(12.5)	704	(14.0)	2,833	(29.2)	16,959	(29.1)
Immunosuppressants	198	(1.7)	631	(0.9)	23	(2.3)	48	(0.8)	11	(1.3)	31	(0.6)	164	(1.7)	552	(0.9)
Bisphosphonates	743	(6.4)	3,365	(4.9)	49	(5.0)	188	(3.2)	7	(0.8)	56	(1.1)	687	(7.1)	3,121	(5.4)
RAAS inhibitors	3,492	(30.3)	18,668	(27.0)	265	(26.8)	1,329	(22.4)	130	(15.5)	712	(14.2)	3,097	(31.9)	16,627	(28.5)
Platelet inhibitors	2,449	(21.2)	15,283	(22.1)	127	(12.9)	960	(16.2)	49	(5.8)	394	(7.8)	2,273	(23.4)	13,929	(23.9)
Beta blockers	2,080	(18.0)	11,790	(17.0)	125	(12.7)	747	(12.6)	77	(9.2)	433	(8.6)	1,878	(19.3)	10,610	(18.2)
Calcium channel blockers	2,356	(20.4)	12,120	(17.5)	179	(18.1)	854	(14.4)	73	(8.7)	410	(8.2)	2,104	(21.7)	10,856	(18.6)
Diuretics	3,345	(29.0)	17,240	(24.9)	212	(21.5)	1,086	(18.3)	82	(9.8)	445	(8.9)	3,051	(31.4)	15,709	(27.0)
Organic nitrates	488	(4.2)	3,388	(4.9)	20	(2.0)	183	(3.1)	7	(0.8)	82	(1.6)	461	(4.7)	3,123	(5.4)

Abbreviations: BMI = body mass index; COPD = chronic obstructive pulmonary disease; NJR = National Joint Registry; NSAID = non-steroidal anti-inflammatory drug; RAAS = renin-angiotensin-aldosterone-system; SD = standard deviation; THR = total hip replacement.

### Bias-analysis (NJR Cohort)

Table 2 shows a healthy user effect during the first six months after THR surgery. During the period of time immediately after the THR, we observed a decreased risk of any cancer in patients with THR (adjusted RR 0.74; 95% CI 0.57–0.95) compared with matched referent subjects. There was a trend of differences in cancer risk between the different types of bearing surfaces during the first six months after THR surgery.

**Table 2:** Bias-analysis: Relative rates of types of cancer (recorded in the CPRD)<six months after total hip replacement (recorded in NJR, stratified by bearing surface type), compared with matched controls without THR surgery, and patients with stemmed metal-on-metal total hip replacements (NJR cohort).

					Compared with no THR	
	THR patients		Control patients		Adjusted relative	
	n cases	rate	n cases	rate	rate (95% CI) (a)	
All total hip replacements						
Any cancer	75	1.33	611	1.82	0.74	(0.57–0.95)
By bearing surface type						
Stemmed metal-on-metal	3	0.84	29	0.94	0.80	(0.23–2.76)
Resurfacing	1	0.26	17	0.62	0.39	(0.05–3.12)
Other bearing surfaces	71	1.51	565	1.99	0.75	(0.59–0.96)
Haematological cancer	10	0.18	39	0.12	1.41	(0.67–2.98)
Malignant melanoma	1	0.02	19	0.06	0.26	(0.03–1.99)
Prostate cancer	12	0.21	62	0.18	1.14	(0.59–2.20)
Renal cancer	5	0.09	39	0.12	0.82	(0.31–2.16)
Other cancer	47	0.83	452	1.34	0.63	(0.46–0.86)

Abbreviations: CI = confidence interval; CPRD = Clinical Practice Research Datalink; n = number; NJR = National Joint Registry; THR = total hip replacement.

Rates are number of events per 100 person years. (a) Adjusted for small-area socioeconomic status, smoking status, use of alcohol, body mass index, a history of hypertension, chronic obstructive pulmonary disease (COPD), coronary artery disease (CAD), and a prescribing in the 6 months before of NSAIDs or aspirin, oestrogen containing drugs, oral glucocorticoids, calcium/vitamin D supplements, glucose lowering agents, statins, immunosuppressive agents, bisphosphonates, renin-angiotensin-aldosterone-system (RAAS) inhibitors, platelet inhibitors, beta blockers, calcium channel blockers, diuretics, and organic nitrates.

### Association between Hip Replacement and Cancer Risk

The risk of cancer was not increased in THR patients (any bearing surface type) compared with matched referent subjects in any of the three study cohorts and using any of the sources for cancer outcomes (Table 3). In the CPRD THR cohort, the adjusted RR was 0.76 (95% CI 0.73–0.79) using the CPRD for cancer outcomes, 0.73 (95% CI 0.68–0.77) using HES for cancer outcomes and 0.70 (95% CI 0.64–0.75) using national death certificates. These results closely resembled those of the bias analysis. This implies that patients undergoing THR surgery are healthier at baseline, probably reflecting the selection for surgical fitness. This emphasizes the need for careful timing analyses, rather than overall associations. Similar trends were seen across all databases. Compared with stemmed metal-on-metal hip replacements (NJR cohort), risk of cancer was similar with hip resurfacing (adjusted RR 0.69; 95% CI 0.39–1.22) or other types of bearing surfaces (adjusted RR 0.96; 95% CI 0.64–1.43).

**Table 3:** Relative rates of cancer (any type as recorded in the CPRD, HES or ONS) during the total follow-up period in patients with and without total hip replacements (as recorded in the CPRD, HES or NJR).

						Age-, sex-, calendar		Adjusted	
		Total hip replacement		Controls		year-adjusted relative		relative rate	
Cohort source	Cancer source	n cases	rate	n cases	rate	rate (95% CI)		(95% CI) (a)	
CPRD	CPRD	3,752	1.62	25,786	2.11	0.77	(0.74–0.79)	0.76	(0.73–0.79)
	HES	1,417	1.26	10,166	1.73	0.73	(0.69–0.77)	0.73	(0.68–0.77)
	ONS	794	0.69	6,058	1.00	0.69	(0.64–0.74)	0.70	(0.64–0.75)
HES	CPRD	1,640	1.69	10,614	1.99	0.85	(0.81–0.89)	0.84	(0.80–0.89)
	HES	1,135	1.37	7,488	1.64	0.83	(0.78–0.89)	0.81	(0.76–0.87)
	ONS	640	0.75	4,469	0.95	0.79	(0.72–0.86)	0.78	(0.72–0.85)
NJR	CPRD	721	1.69	4,563	1.89	0.89	(0.83–0.96)	0.89	(0.82–0.97)
	HES	399	1.34	2,701	1.59	0.84	(0.76–0.93)	0.83	(0.74–0.93)
	ONS	190	0.63	1,395	0.80	0.76	(0.67–0.90)	0.80	(0.68–0.94)

Abbreviations: CI = confidence interval; CPRD = Clinical Practice Research Datalink; HES = Hospital Episode Statistics; n = number; NJR = National Joint Registry; ONS = Office for National Statistics (death certificates).

Rates are number of events per 100 person years.

(a) Adjusted for confounders as shown in Table 2.

Table 4 displays the cumulative incidence rates of cancer over time, stratified by bearing surface type, gender and age (NJR cohort). Overall, we did not find an increased excess rate of cancer in patients with metal-on-metal hip devices as compared to other bearing surface types. Although there were higher excess rates in specific patient groups (e.g. 80+ years males), the excess rates did not increase over time, suggesting confounding rather than a true causal relationship.

**Table 4:** Cumulative incidence (%) of any cancer (as recorded in CPRD) after total hip replacement by bearing surface type, gender, and age categories gender, and age categories (NJR cohort).

	Cumulative incidence of any cancer (95% confidence interval)				
	≤1 years	≤2 years	≤3 years	≤4 years	≤5 years
**Males**					
** Age 18–59**					
All total hip replacements	0.6 (0.3–0.9)	1.2 (0.8–1.6)	1.7 (1.3–2.1)	2.4 (2.0–2.8)	3.0 (2.6–3.4)
Resurfacing	0.4 (0.0–1.4)	0.8 (0.0–1.9)	1.3 (0.1–2.5)	1.7 (0.4–3.0)	2.2 (0.8–3.6)
Stemmed metal-on-metal	0.8 (0.0–2.1)	1.6 (0.2–3.0)	2.4 (0.9–3.9)	3.5 (1.8–5.2)	4.2 (2.4–6.0)
Other bearing surfaces	0.6 (0.2–1.0)	1.3 (0.9–1.7)	1.9 (1.5–2.3)	2.6 (2.1–3.1)	3.3 (2.8–3.8)
** Age 60–79**					
All total hip replacements	1.7 (1.2–2.2)	3.4 (2.9–3.9)	5.1 (4.5–5.7)	6.9 (6.3–7.5)	8.7 (8.0–9.4)
Resurfacing	1.4 (0.0–3.1)	3.0 (1.2–4.8)	4.5 (2.6–6.4)	6.3 (4.2–8.4)	8.0 (5.7–10.3)
Stemmed metal-on-metal	1.6 (0.0–3.2)	3.3 (1.5–5.1)	4.9 (3.0–6.8)	7.2 (5.1–9.3)	8.4 (6.2–10.6)
Other bearing surfaces	1.8 (1.2–2.4)	3.5 (2.9–4.1)	5.2 (4.6–5.8)	7.0 (6.3–7.7)	8.8 (8.1–9.5)
** Age 80+**					
All total hip replacements	2.3 (1.0–3.6)	4.6 (3.3–5.9)	6.6 (5.2–8.0)	8.7 (7.2–10.2)	10.7 (9.1–12.3)
Resurfacing	2.8 (0.0–8.1)	5.9 (0.3–11.5)	8.2 (2.5–13.9)	12.1 (5.7–18.5)	15.2 (8.3–22.1)
Stemmed metal-on-metal	2.6 (0.0–7.2)	5.2 (0.3–10.1)	7.6 (2.5–12.7)	10.7 (5.1–16.3)	12.5 (6.7–18.3)
Other bearing surfaces	2.4 (1.0–3.8)	4.6 (3.1–6.1)	6.7 (5.2–8.2)	8.7 (7.1–10.3)	10.7 (8.9–12.5)
**Females**					
** Age 18–59**					
All total hip replacements	0.4 (0.1–0.7)	0.8 (0.5–1.1)	1.2 (0.9–1.5)	1.7 (1.4–2.0)	2.1 (1.7–2.5)
Resurfacing	0.4 (0.0–1.4)	0.8 (0.0–1.9)	1.2 (0.1–2.3)	1.7 (0.4–3.0)	2.2 (0.8–3.6)
Stemmed metal-on-metal	0.6 (0.0–1.7)	1.3 (0.1–2.5)	1.9 (0.6–3.2)	2.8 (1.3–4.3)	3.3 (1.7–4.9)
Other bearing surfaces	0.4 (0.1–0.7)	0.9 (0.6–1.2)	1.3 (1.0–1.6)	1.8 (1.4–2.2)	2.3 (1.9–2.7)
** Age 60–79**					
All total hip replacements	1.2 (0.8–1.6)	2.4 (2.0–2.8)	3.6 (3.1–4.1)	4.9 (4.4–5.4)	6.2 (5.6–6.8)
Resurfacing	1.4 (0.0–3.1)	3.1 (1.3–4.9)	4.6 (2.6–6.6)	6.4 (4.3–8.6)	8.1 (5.8–10.4)
Stemmed metal-on-metal	1.3 (0.0–2.8)	2.6 (1.0–4.2)	4.0 (2.3–5.7)	5.8 (3.9–7.7)	6.8 (4.8–8.8)
Other bearing surfaces	1.2 (0.7–1.7)	2.4 (1.9–2.9)	3.6 (3.1–4.1)	4.8 (4.2–5.4)	6.1 (5.5–6.7)
** Age 80+**					
All total hip replacements	1.7 (0.6–2.8)	3.3 (2.2–4.4)	4.8 (3.6–6.0)	6.4 (5.1–7.7)	7.9 (6.5–9.3)
Resurfacing	–	–	–	–	–
Stemmed metal-on-metal	2.0 (0.0–6.1)	4.2 (0.0–8.6)	6.2 (1.6–10.8)	8.8 (3.7–13.9)	10.3 (4.9–15.7)
Other bearing surfaces	1.6 (0.4–2.8)	3.1 (1.9–4.3)	4.6 (3.3–5.9)	6.1 (4.7–7.5)	7.6 (6.1–9.1)

Abbreviations: CPRD = Clinical Practice Research Datalink.

### Patterns and Timing of Cancer Risk

**Figure 1:**
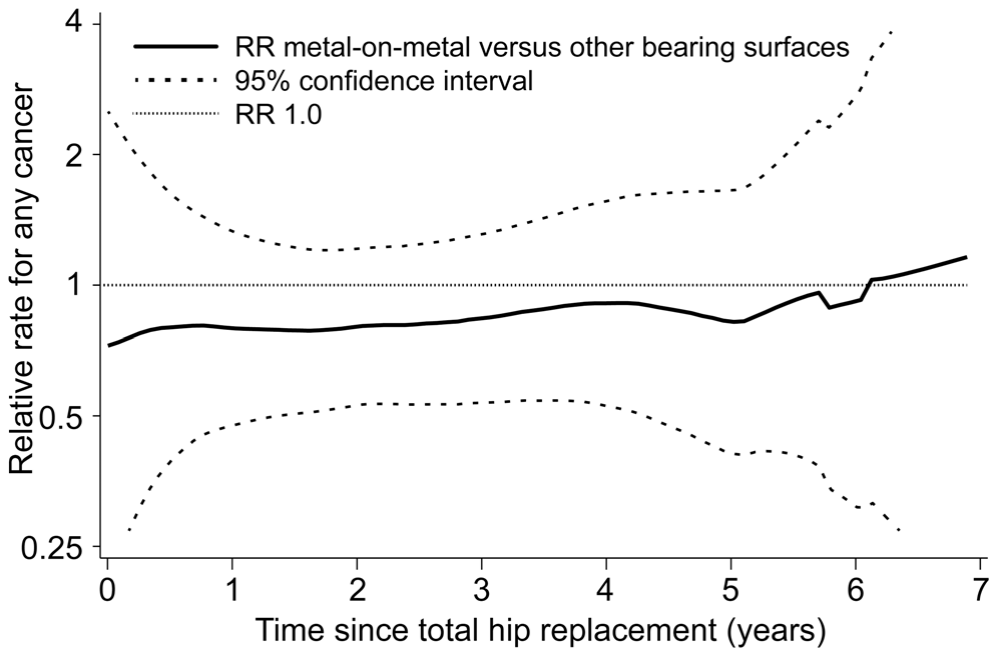
Crude RR of cancer over time in CPRD (and 95% CI) in patients with a metal-on-metal THR compared to patients with another bearing surface THR (as recorded in NJR). Abbreviations: CI = confidence interval; CPRD = Clinical Practice Research Datalink; NJR = National Joint Registry.

Table 5 shows the risk of cancer over time within THR patients and matched referent subjects, stratified by type of cancer (CPRD cohort). The risk of cancer increased over time in both THR patients and referent subjects. Figure 1 shows that risk of cancer in patients with metal-on-metal hip devices remained constant over time compared to individuals with hip implants of other bearing surface types (NJR cohort).

**Table 5:** Relative rates of types of cancer (as recorded in the CPRD) over time (CPRD cohort).

	Total hip replacement			Controls			
Time period(months)	n cases	rate	AdjustedRR (95% CI)	n cases	rate	AdjustedRR (95% CI)	
Any cancer							
<6	290	1.31	reference	2,722	2.06	reference	
6–24	870	1.48	1.13 (0.99–1.29)	6,903	2.04	0.97 (0.93–1.02)	
25–60	1,260	1.56	1.17 (1.03–1.33)	9,191	2.13	0.96 (0.92–1.01)	
>60	1,332	1.90	2.10 (1.84–2.41)	6,970	2.18	1.31 (1.25–1.38)	
Haematological cancer							
<6	32	0.14	reference	203	0.15	reference	
6–24	82	0.14	0.95 (0.63–1.43)	496	0.15	0.93 (0.79–1.10)	
25–60	77	0.10	0.63 (0.42–0.96)	638	0.15	0.88 (0.75–1.03)	
>60	94	0.13	1.31 (0.85–2.04)	462	0.14	1.08 (0.91–1.29)	
Malignant melanoma							
<6	4	0.02	reference	91	0.07	reference	
6–24	31	0.05	2.89 (1.02–8.19)	238	0.07	1.01 (0.79–1.28)	
25–60	52	0.06	3.38 (1.22–9.38)	270	0.06	0.86 (0.67–1.09)	
>60	46	0.07	4.02 (1.41–11.52)	236	0.07	1.06 (0.82–1.37)	
Prostate cancer							
<6	42	0.19	reference	294	0.22	reference	
6–24	116	0.20	1.04 (0.73–1.48)	707	0.21	0.92 (0.81–1.06)	
25–60	142	0.18	0.91 (0.64–1.29)	969	0.22	0.96 (0.84–1.10)	
>60	163	0.23	1.93 (1.34–2.80)	777	0.24	1.64 (1.42–1.90)	
Renal cancer							
<6	23	0.10	reference	180	0.14	reference	
6–24	61	0.10	0.99 (0.61–1.60)	538	0.16	1.15 (0.97–1.37)	
25–60	93	0.11	1.06 (0.67–1.67)	712	0.16	1.15 (0.98–1.36)	
>60	88	0.13	1.68 (1.03–2.76)	501	0.16	1.53 (1.28–1.84)	
Other cancer							
<6	189	0.86	reference	1,954	1.48	reference	
6–24	580	0.99	1.16 (0.98–1.36)	4,924	1.45	0.97 (0.92–1.02)	
25–60	896	1.11	1.28 (1.09–1.50)	6,602	1.54	0.96 (0.91–1.01)	
>60	941	1.34	2.30 (1.94–2.72)	4,994	1.57	1.29 (1.22–1.36)	

Abbreviations: CPRD = Clinical Practice Research Datalink; CI = confidence interval; RR = relative rate.

## Discussion

This study found that patients with metal-on-metal THR were not at increased risk of cancer compared to individuals with hip implants of other bearing surface types. The results of the patterns of cancer risk over time did not find increases of cancer risk over time. There were substantial differences in baseline characteristics between patients who received metal-on metal hip implants and those with other bearing surfaces. Elderly patients and patients with chronic conditions were less likely to receive a metal-on-metal THR and these factors were found to be associated with the risk of cancer.

### Comparison with Other Studies

Our findings are in line with two recent observational studies investigating the risk of cancer in patients with metal on metal hip replacement [8], [11]. Similar to our study, these British and Finnish studies could not find an increased risk of cancer and reported incidence rates that were consistent with our study. However, these previous studies did not have detailed information on risk factors, with only a limited comparison of baseline characteristics between the different types of THR. The present study found that there is strong evidence for confounding between the different types of THR. A Finnish cohort study, comprising 2,164 patients with a mean follow-up of 17 years, showed an increased cancer-related mortality rate in patients with metal-on-metal hip implants (standardised mortality ratio [SMR] of 0.97) compared to individuals with metal-on-polyethylene prostheses (SMR of 0.76) [26]. However, as they did not look at incident cancer events, this may well represent confounding by contraindication: individuals with metal-on-polyethylene prostheses are in general older (and may already have developed cancer) and cancer is a relative contraindication for THR. Moreover, life expectancy may be shortened in (prevalent) cancer patients and surgeons may therefore decide not to perform a major elective surgery (such as THR) in these patients. An alternative explanation of the discrepant results is that the length of follow-up (up to 7 years) was shorter in the present study than that in the Finnish study. It has been shown in animal studies that there may be a long latency in the development of tumours following exposure to metal compounds, which may translate to a latency of 10 years in humans [27]. Most other observational studies could not differentiate between bearing surface types [7]. A meta-analysis including nine of these studies compared risk of cancer in patients with total joint arthroplasty with age- and gender-specific expected cancer rates [21]. In line with our findings, the authors could not find an overall increased risk of any cancer.

This study demonstrates the importance of linkages between different electronic health records for health surveillance monitoring. Whilst the NJR has excellent data on the type of prosthesis, it contains limited data on clinical patient related variables and co-morbidities. In our study, we have shown substantial differences in these clinical variables between prosthesis types, and need to be considered as confounding factors. CPRD does have very extensive information on these variables, as well as comprehensive data on drug and health service utilisation but does not contain detailed surgical information. The linkage of NJR and CPRD does provide an efficient tool for long-term safety monitoring of joint replacements.

### Strengths and Limitations

The major strength of this study is the linkage between NJR and CPRD which provided detailed information on bearing surface type (NJR) as well as clinical risk factors for cancer (CPRD). Our study had a reasonable sample size and cancer outcomes were obtained through three independently collected databases. A limitation is the lack of information on underlying disease severity (which may have influenced cancer risk) and other potential confounders. Referent subjects were not matched on osteoarthritis (the main indication for THR), which is associated with a decreased risk of cancer [28]. Although this may have underestimated our observed relationship, this is likely to be constant over time and should not have had an impact on the patterns of cancer risk over time. Moreover, this should not have influenced our comparison between bearing surface types, as they should be more or less homogenous with respect to osteoarthritis. We may not be able to extrapolate our findings to long-term situations as some cancers are known to have a prolonged latency since start of exposure. In addition, as explained above, there may be a long latency in the development of tumours following metal exposure, although this has only been based on animal studies [27].

### Conclusions and Study Implications

This study provides reassuring results with respect to the possible signal of increased risks of cancer with metal-on-metal hip replacements. We could not find an elevated risk of cancer with metal-on-metal hip implants and the analyses of cancer risk over time did not support a causal relationship. There were substantial differences in baseline characteristics between the different types of THR complicating the interpretation of a direct comparison between bearing surfaces. The analyses in this study will need to be repeated in the future longer follow-up data, in particular as cancer latency may be prolonged for specific cancer types and following metal exposure.
